# Supplementary material for: Persistent virus-specific and clonally expanded antibody-secreting cells respond to induced self-antigen in the CNS
Source: Acta Neuropathol. 2023 Jan 25;145(3):335–55. doi: 10.1007/s00401-023-02537-5 (PMC9925600; doi:10.1007/s00401-023-02537-5)
Supplement: Supplementary file 2 — Supplementary file2 (PDF 5753 KB) [file 401_2023_2537_MOESM2_ESM.pdf]

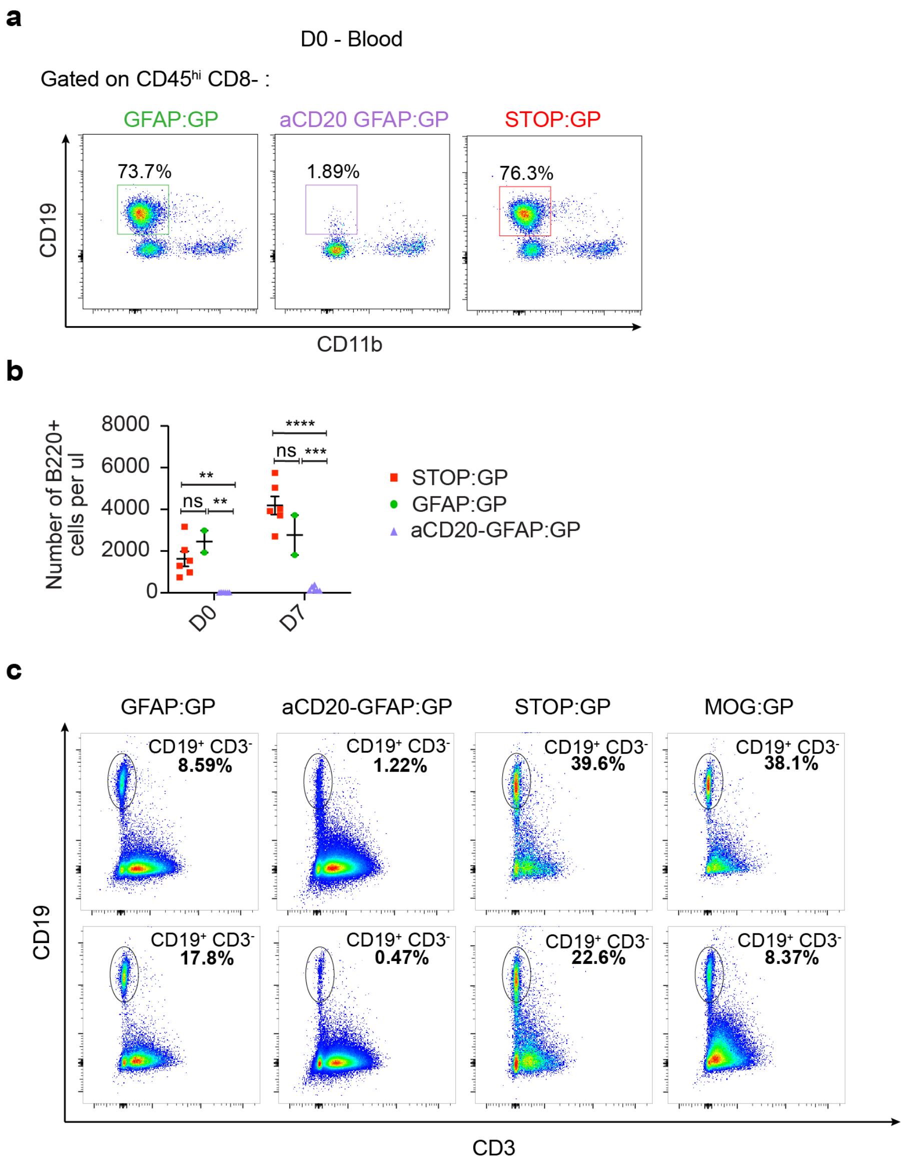

Supplementary figure 1. Flow cytometry of murine CNS B cells. a. Flow cytometry plots of CD45hi CD8- CD11b CD19+ cells in the blood at day 0 of tamoxifen administration in indicated groups. Numbers represent the percentage of positive cells. b. Number of B220+ cells in blood upon TAM administration (day 0) and on the day of sacrifice (day 7) determined by flow cytometry. Symbols represent one individual mouse, and barsdata represent means ± SEM. *$P < 0.05$; ns, not significant; two-way ANOVA followed by Tukey's multiple comparisons test c. Flow cytometry dot plot analysis of sorted $CD19^{+}/CD3^{-}$ B cell populations per mouse for each experimental group for remaining mice not shown in figure 1f.

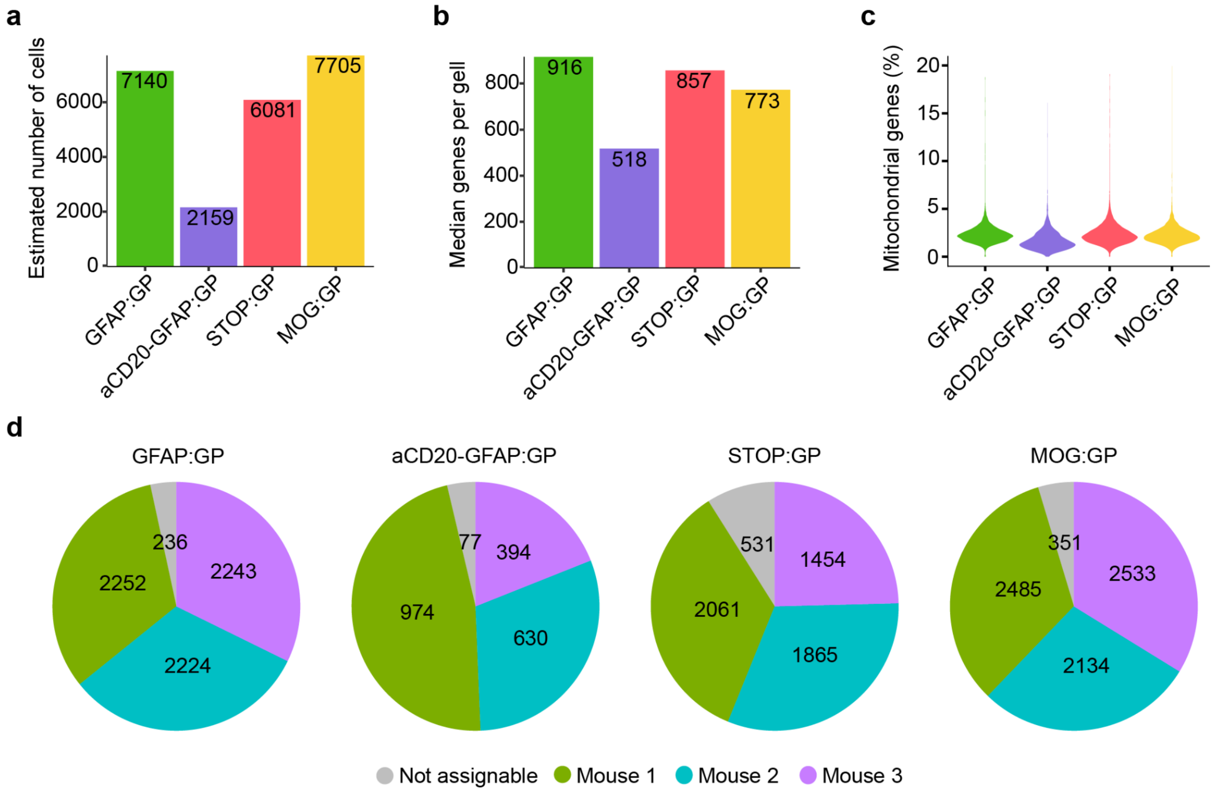

Supplementary figure 2. General statistics following single-cell sequencing. a. Number of cell barcodes in gene expression (GEX) sequencing libraries b. Number of unique genes per cell. c. Average number of mitochondrial reads per cell. d. Number of cells per hashing barcodes.

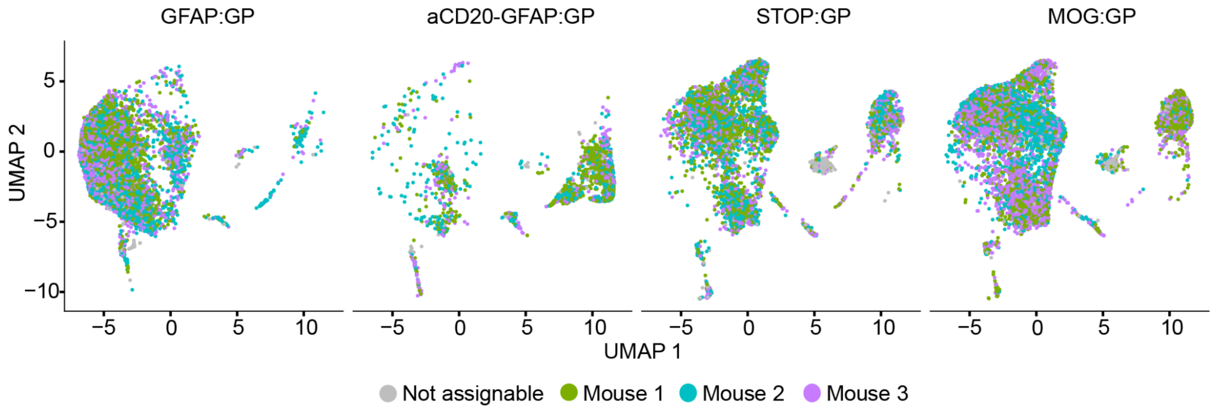

Supplementary figure 3. Uniform manifold approximation projection (UMAP) split by sample and colored by mouse-specific DNA barcode demonstrating biological reproducibility.

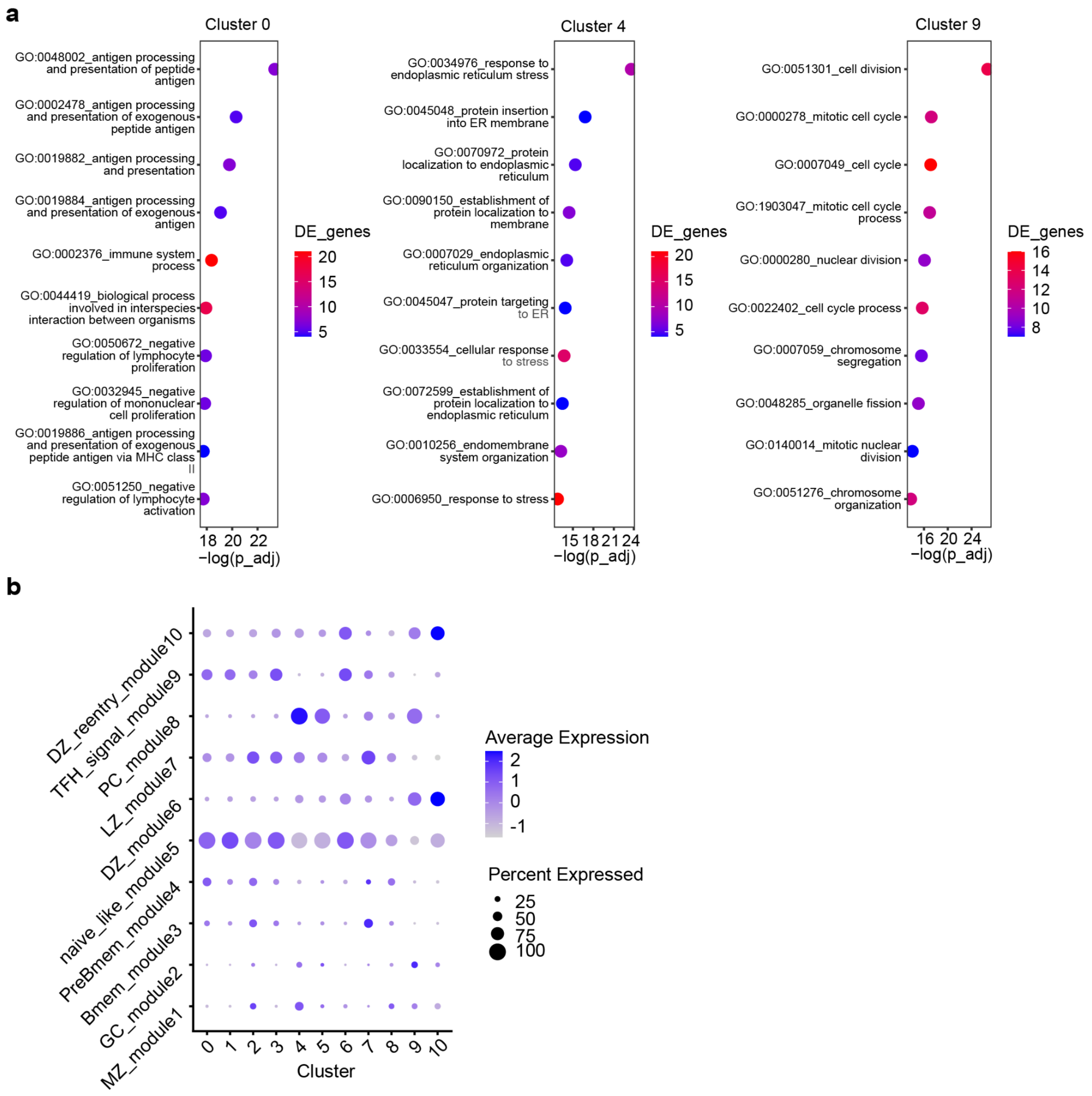

Supplementary figure 4. Gene ontology (GO) and gene modules to classify B cell phenotypes. a. GO term enrichment of the 10 most upregulated genes from transcriptional clusters 0, 4 and 9 based on average log fold change. The color of each dot corresponds to the adjusted p-value and the size corresponds to the number of genes. Ratio corresponds to the number of differentially expressed genes relative to the number of total genes corresponding to each GO term. b. Dottile plot showing B cell subset assignment across all clusters based on expression of genes defining B cell phenotypes. The intensity of each dot corresponds to the average expression of all cells within a given cluster and the size corresponds to the percentage of cells with detectable gene expression.

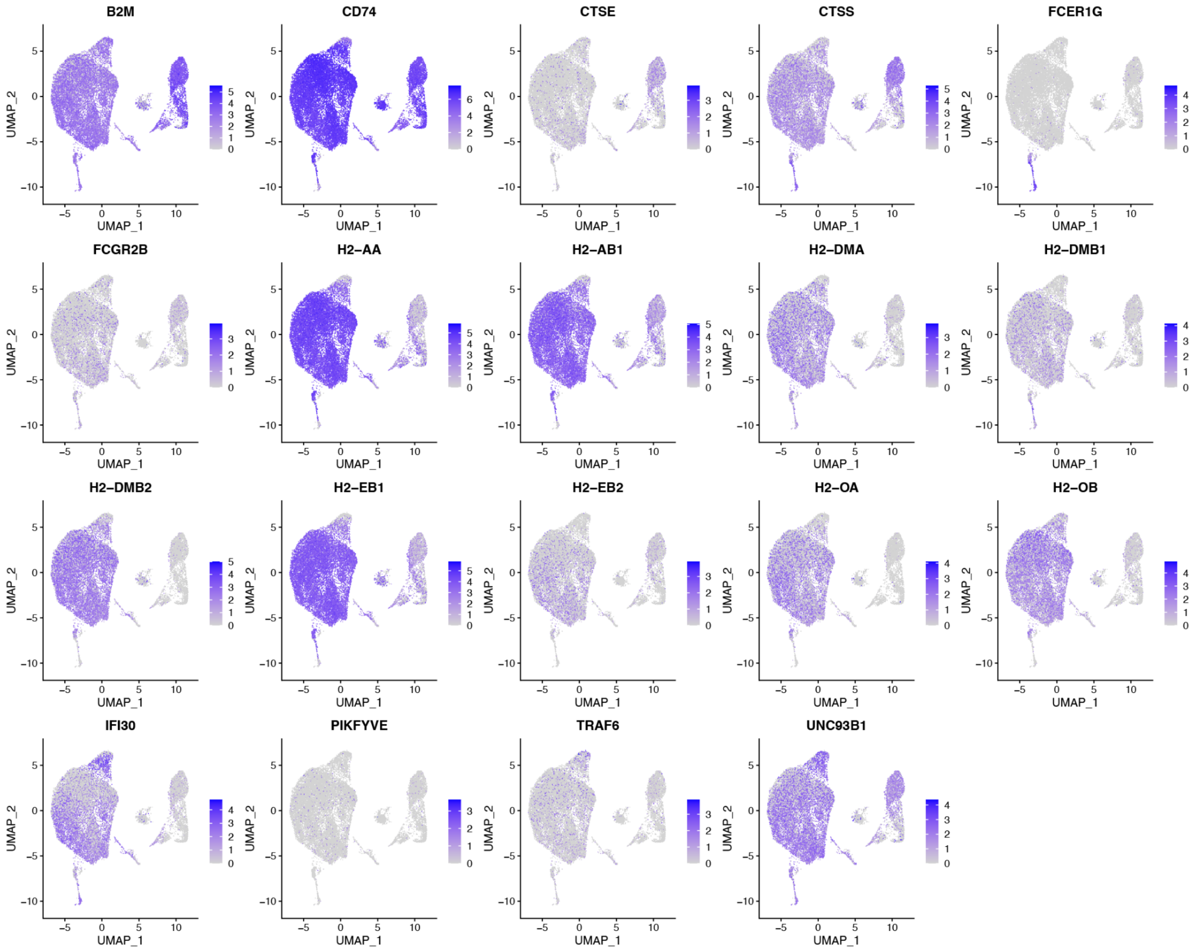

Supplementary figure 5. Gene expression of select genes associated with class II antigen presentation.

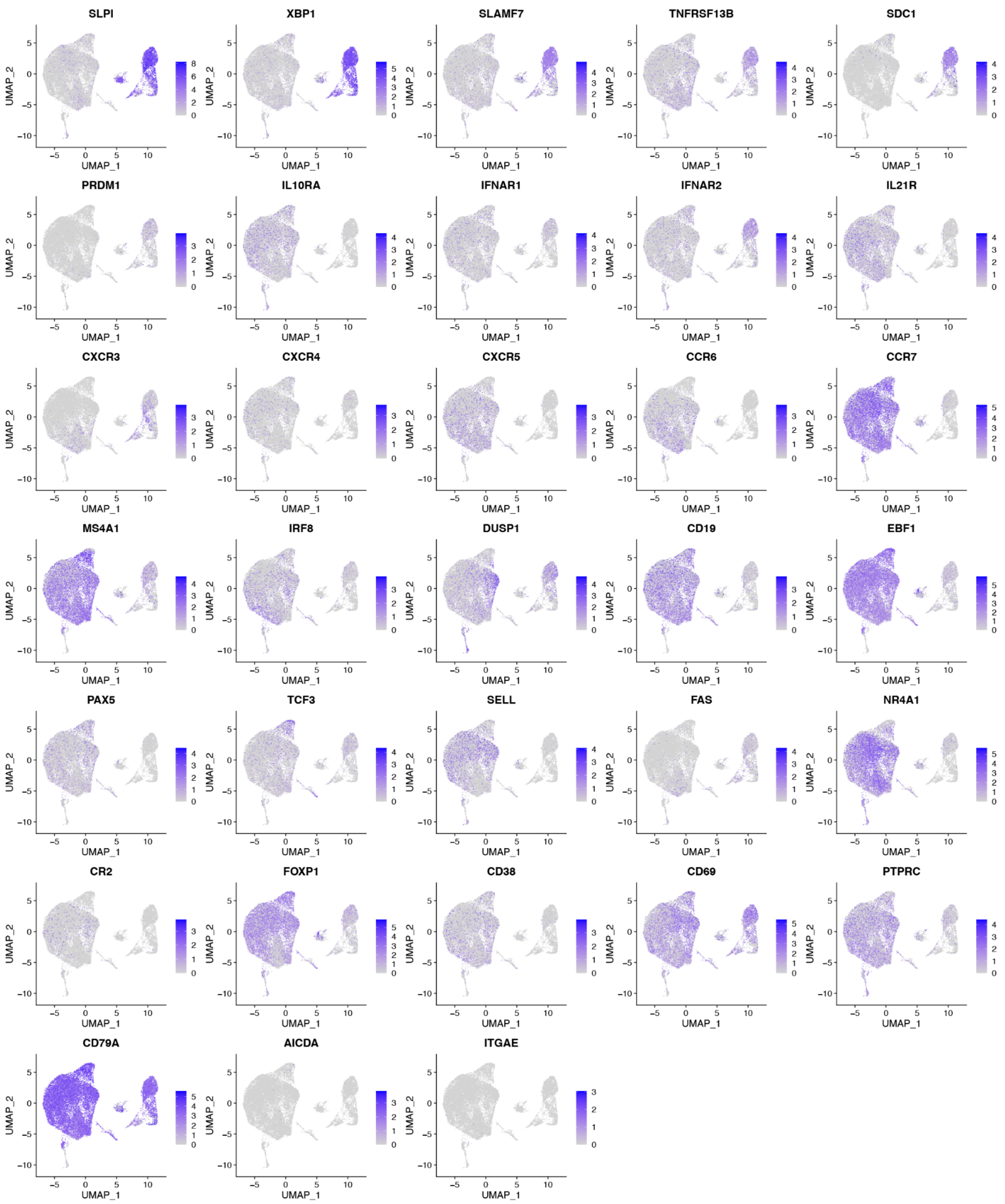

Supplementary figure 6. Gene expression of select genes for all cells from all conditions.

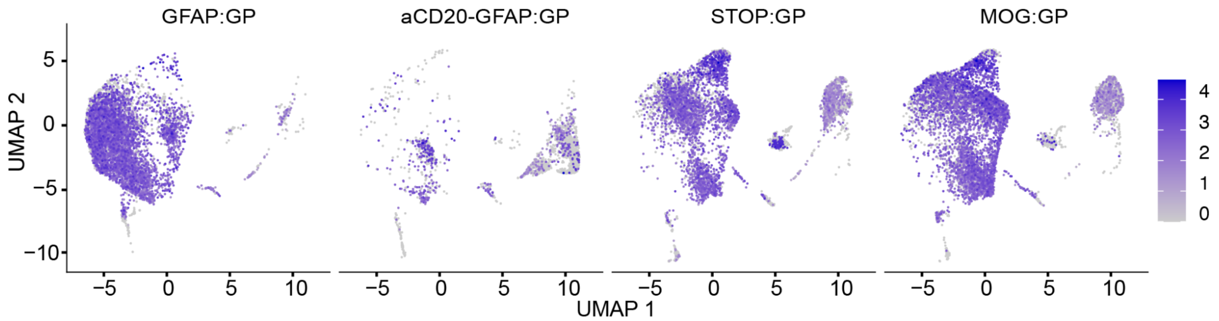

Supplementary figure 7. Gene expression of *CD20* (*MS4A1*) for all cells from all conditions.

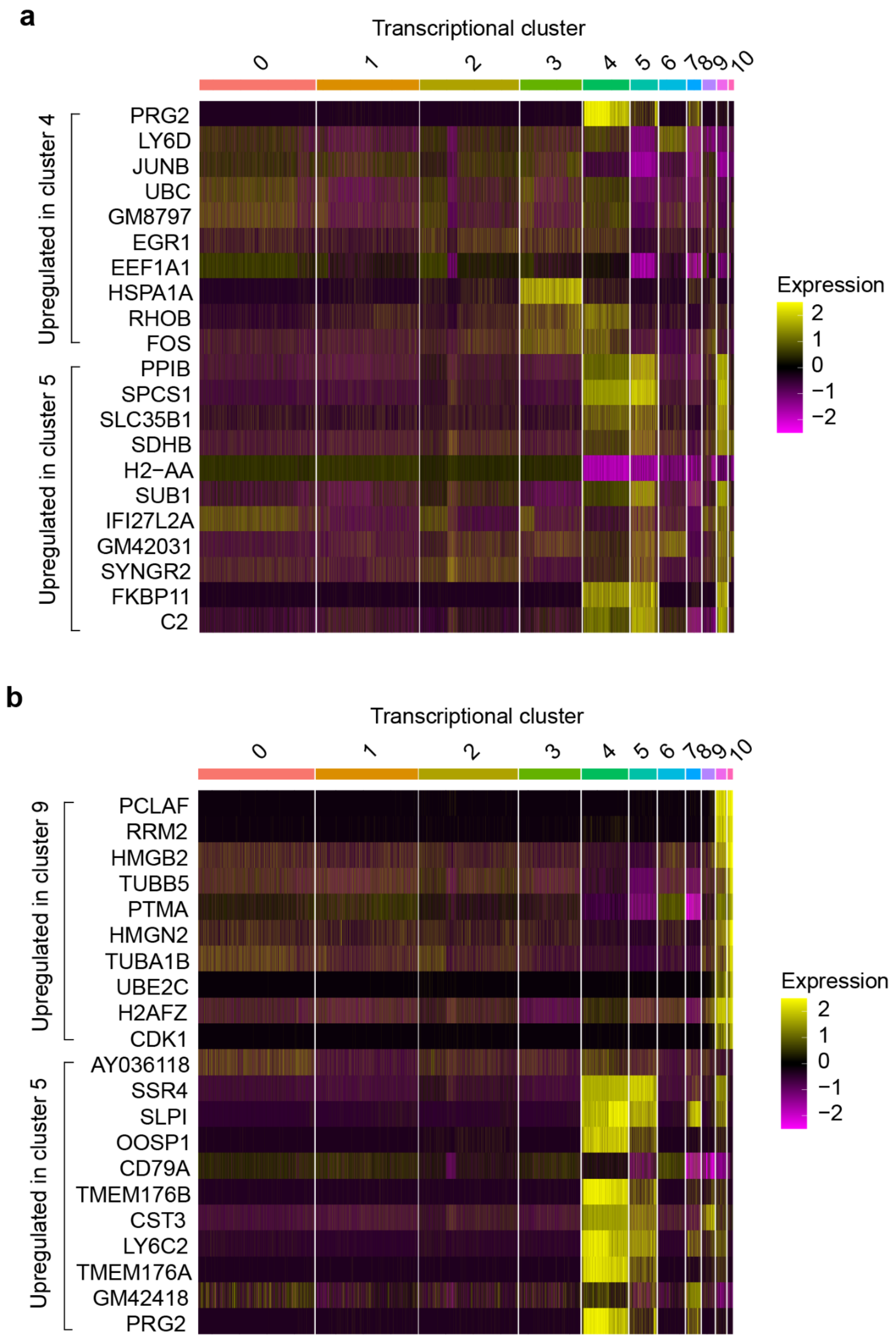

Supplementary figure 8. Distinct gene expression signatures of ASCs located in transcriptional clusters 4, 5, and 9. a. Differentially expressed genes between clusters 4 and 5 ranked by average log-fold change. b. Differentially expressed genes between clusters 9 and 5 ranked by average log-fold change.

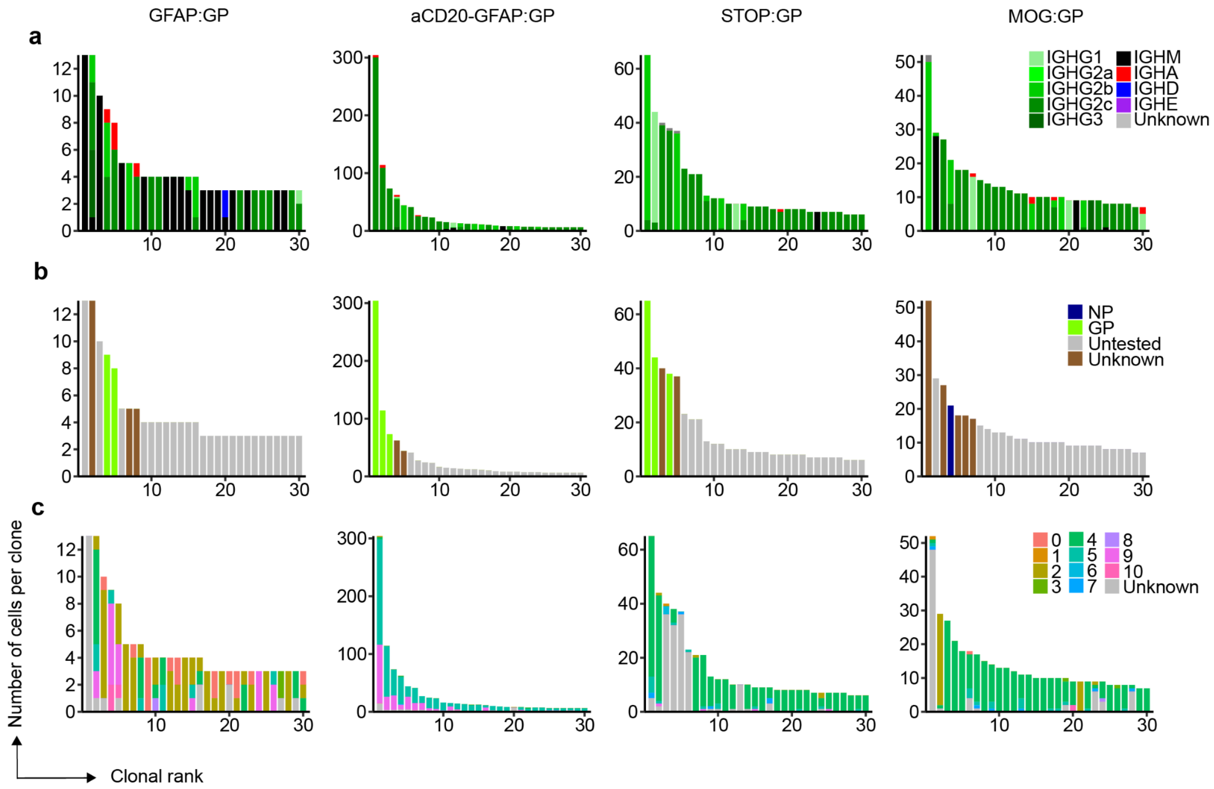

Supplementary figure 9. Isotype, specificity, and transcriptional cluster of the 30 most expanded clones per experimental condition. Clonotyping was performed based on those B cells containing identical CDRH3 + CDRL3 amino acid sequences. Cells or clones were colored based on their a. isotype, b. antigen-specificity (one variant tested per clonal family), or c. transcriptional clustering based on gene expression data.

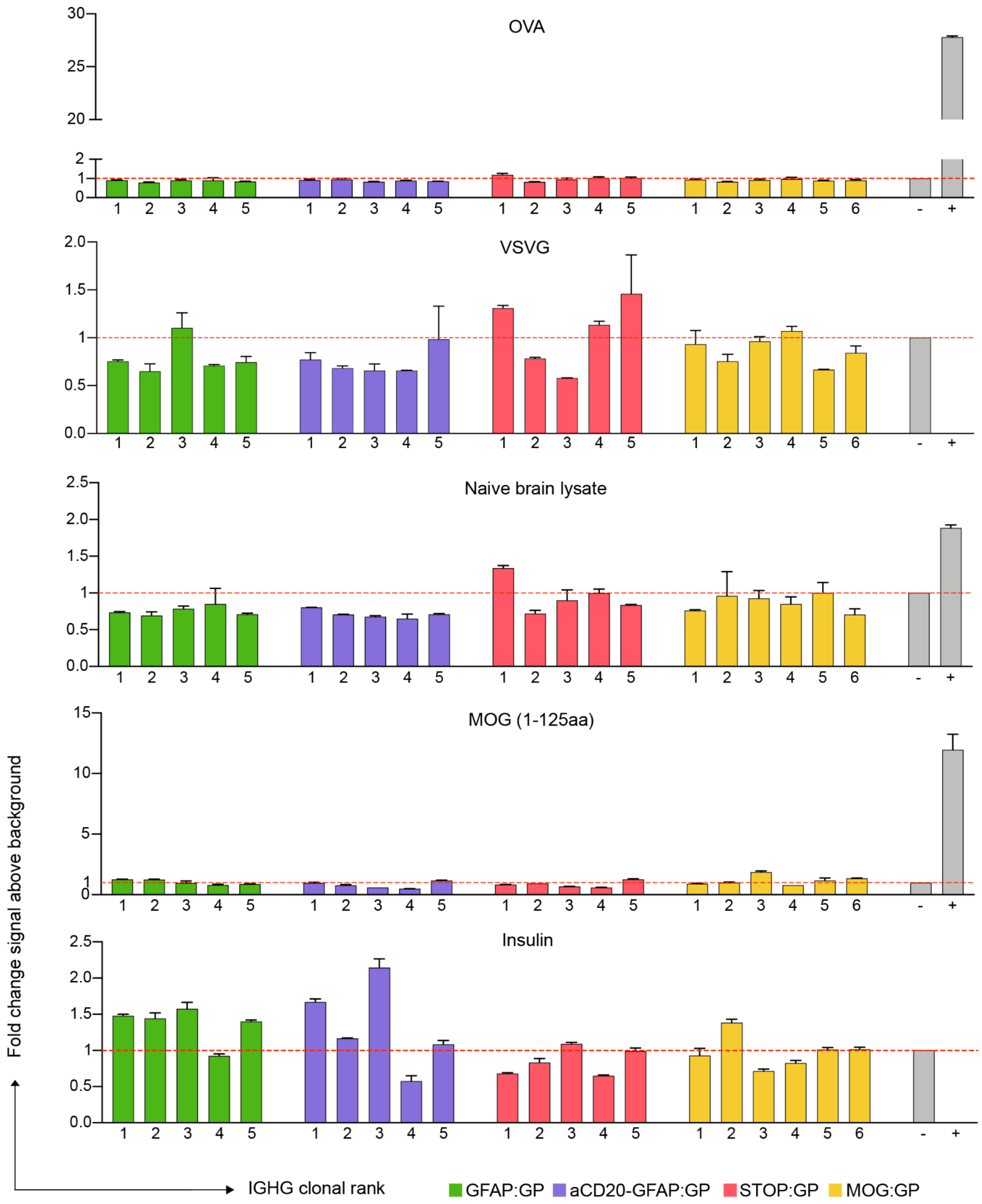

Supplementary figure 10. Antigen specificity of clonally expanded and class-switched ASCs. ELISA signal against ovalbumin (OVA), vesicular stomatitis virus glycoprotein (VSVG) ,naive brain lysate from naive B6 mouse, myelin oligodendrocyte glycoprotein (MOG) protein (amino acid sequence 1-125), and insulin. Clonal rank was determined within each group based on the highest number of cells within each clonotype.

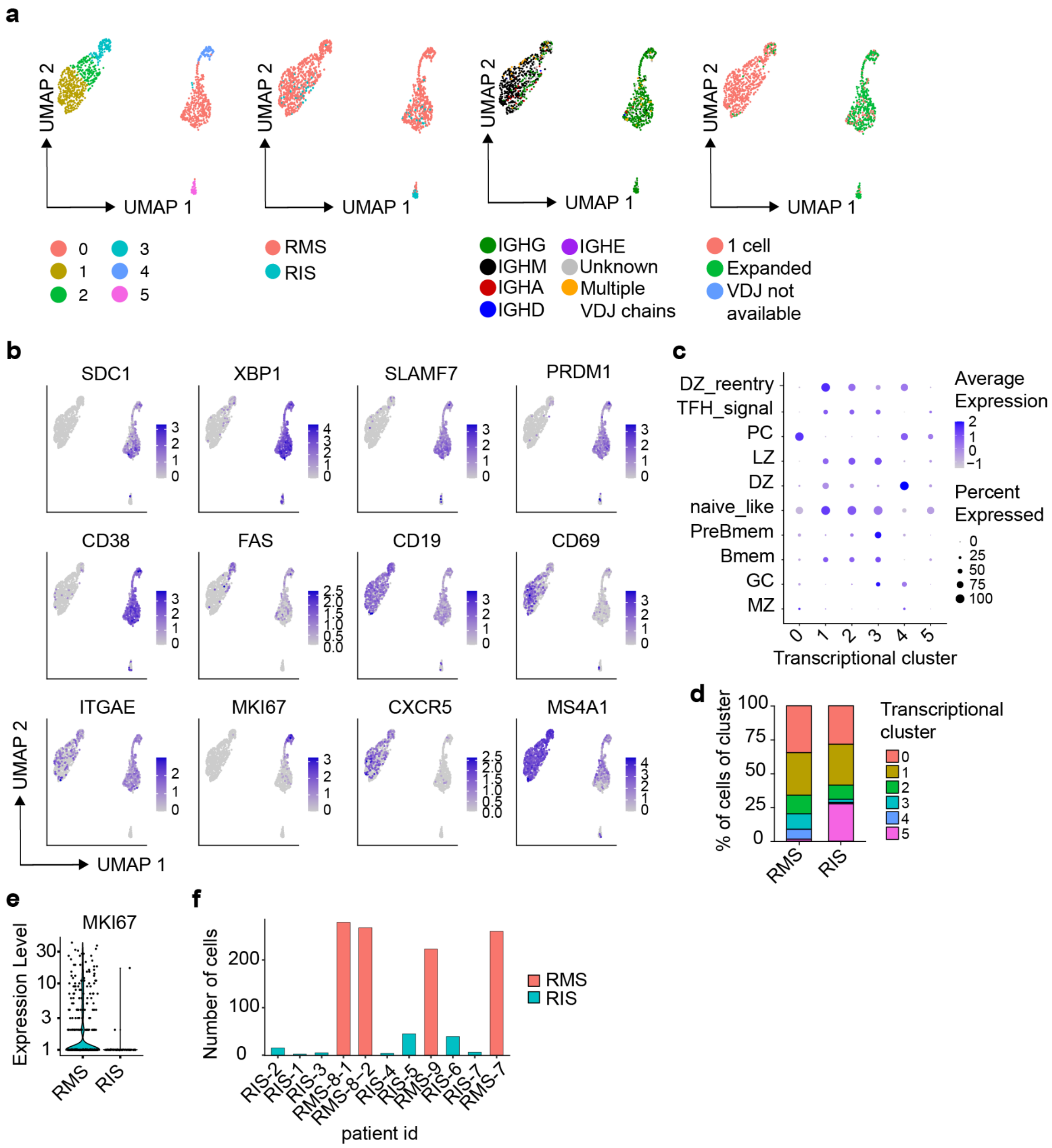

Supplementary figure 11. Transcriptional comparisons between relapsing multiple sclerosis (RMS) and radiologically isolated syndrome (RIS) patients. a. Uniform manifold approximate projection (UMAP) of human RMS and RIS patients colored by transcriptional cluster membership, clinical condition, isotype distribution and cell expansion (from left to right). Expansion corresponds to those cells belonging to clones supported by more than one unique cell barcode. b. Gene expression of select B-cell genes. c. Dottile plot demonstrated B cell subset assignment across all clusters based on expression of previously characterized gene sets. The intensity of each dot corresponds to the average expression of all cells within a given cluster and the size corresponds to the percentage of cells with detectable gene expression. d. Distribution of the transcriptional clusters between the RIS and the Relapse groups. e. Normalized gene expression of *MKI67* in the ASCs of the RMS and RIS groups. f. Number of B cells per patient. Bar color corresponds to clinical condition.

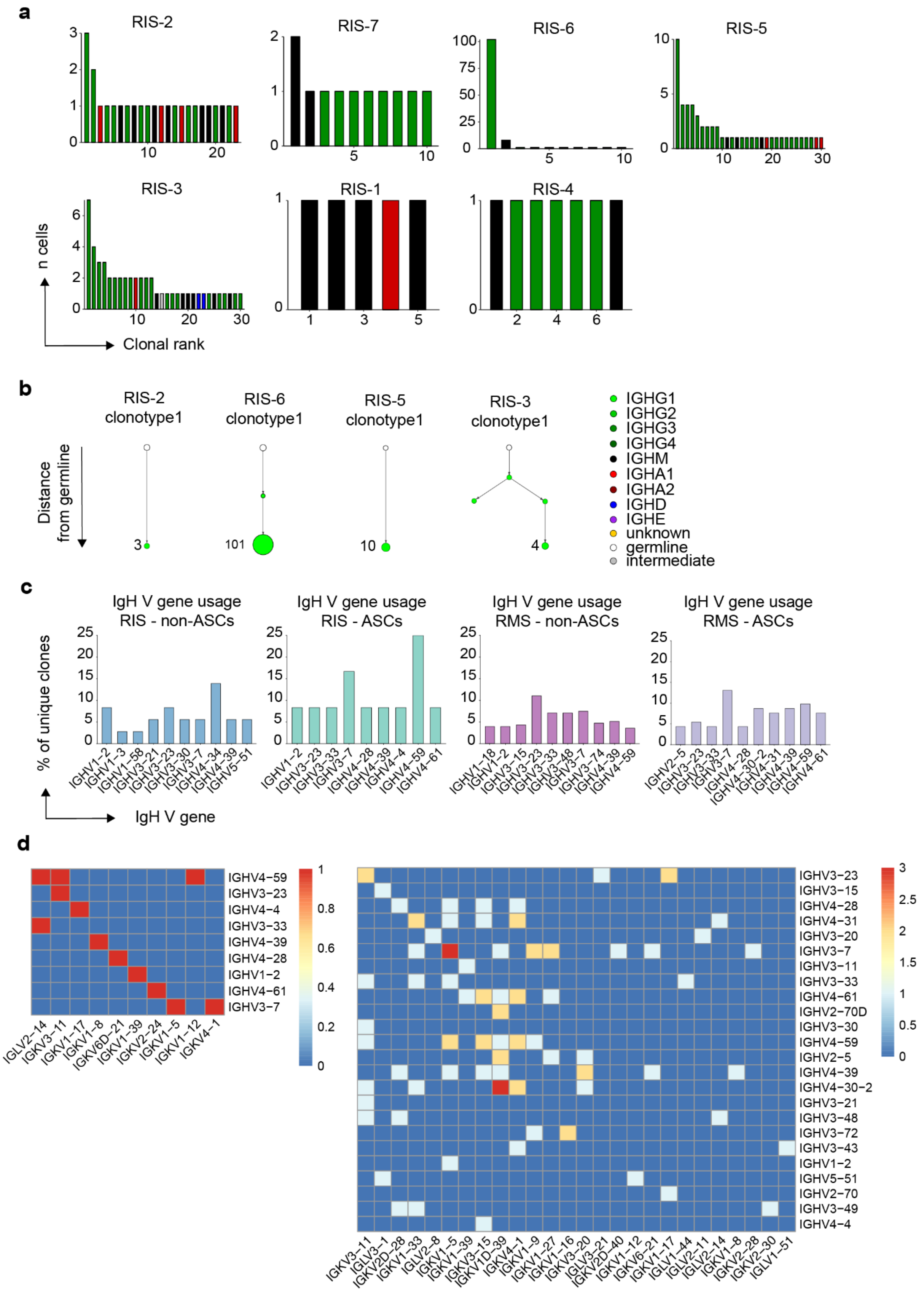

Supplementary figure 12. Repertoire comparisons between relapsing multiple sclerosis (RMS) and radiologically isolated syndrome (RIS) patients. a. Clonal frequencies for the 30 most expanded clones per experimental condition. Clones were determined according to 10x Genomics Cell Ranger's default clonotyping strategy. Color corresponds to isotype as determined in the VDJ

sequencing library. b. Mutational networks of the two most expanded clones per patient. Nodes represent unique antibody variants (combined variable heavy chain [VH] + variable light chain [VL] nucleotide sequences) and edges separate those sequences with the smallest edit distance. Node color corresponds to isotype identity. The size and label of the nodes indicate the number of cells expressing a single full-length antibody variant. Clones were determined according to 10x Genomics Cell Ranger’s default clonotyping strategy and only those cells containing exactly one VH and VL chain were included. The germline node represents the unmutated reference sequence determined by Cellranger. c. Heavy chain V gene usage in non-ASCs and ASCs per clinical condition. d. Heatmap depicting number of clones using a particular heavy chain (HC) and light chain (LC) V gene combination in RIS (left) and RMS patients (right).

Supplementary table 1. Clinical parameters of human samples. Relevant information regarding the human samples used including patient gender, age, date of sampling, date of detection or clinical manifestation, time point of diagnosis and treatment.
